# Effects of rTMS Treatment on Cognitive Impairment and Resting-State Brain Activity in Stroke Patients: A Randomized Clinical Trial

**DOI:** 10.3389/fncir.2020.563777

**Published:** 2020-09-30

**Authors:** Mingyu Yin, Yuanwen Liu, Liying Zhang, Haiqing Zheng, Lingrong Peng, Yinan Ai, Jing Luo, Xiquan Hu

**Affiliations:** ^1^Department of Rehabilitation Medicine, The Third Affiliated Hospital, Sun Yat-sen University, Guangzhou, China; ^2^Department of Radiology, The Third Affiliated Hospital, Sun Yat-sen University, Guangzhou, China

**Keywords:** stroke, cognitive impairment, transcranial magnetic stimulation, amplitude of low frequency fluctuation, functional connectivity

## Abstract

**Background:**

Repetitive transcranial magnetic stimulation (rTMS) has been employed for motor function rehabilitation for stroke patients, but its effects on post-stroke cognitive impairment (PSCI) remains controversial.

**Objective:**

To identify the effects of rTMS intervention on PSCI patients and its potential neural correlates to behavioral improvements.

**Methods:**

We recruited 34 PSCI patients for 20 sessions of 10 Hz rTMS or no-stim control treatments over the left dorsal lateral prefrontal cortex (DLPFC). Cognitive function was evaluated with the Montreal Cognitive Assessment Scale, Victoria Stroop Test, Rivermead Behavior Memory Test, and Activities of Daily Living (ADL) assessed with the Modified Barthel Index. 14 patients received functional MRI scan, a useful non-invasive technique of determining how structurally segregated and functionally specialized brain areas were interconnected, which was reflected by blood oxygenation level–dependent signals. The amplitude of low-frequency fluctuation (ALFF) and functional connectivity (FC) were applied as the analytical approaches, which were used to measure the resting-state brain activity and functional connection.

**Results:**

rTMS improved cognitive functions and ADLs for PSCI patients relative to patients who received no-stim control treatment. The cognitive improvements correlated to increased ALFF of the left medial prefrontal cortex, and increased FC of right medial prefrontal cortex and right ventral anterior cingulate cortex.

**Conclusion:**

10 Hz rTMS at DLPFC could improve cognitive function and quality of life for PSCI patients, which is associated with an altered frontal cortical activity.

**Clinical Registration:**

Chinese Clinical Trial Registry, ChiCTR-IPR-17011908, http://www.chictr.org.cn/index.aspx.

## Introduction

Post-stroke cognitive impairment (PSCI) impairs quality of life in stroke patients. One-third of all stroke patients have varying levels of cognitive impairment and 7% post-stroke patients develop dementia within 1 year (Leys et al., 2005; Sachdev et al., 2006). The core domains for PSCI include executive function, memory, attention, language, and visuospatial function while executive dysfunction and memory disorder are the most common clinical manifestation (Iadecola et al., 2019). With the development of the study of the brain network, cognitive impairments cannot be completely explained by the location after stroke but can be attributed to impairment of brain regions remote to the lesions. For these remote effects, it can be explained that the disruption of neuronal input is vital to the function of that remote brain region or of a certain network (Tuladhar et al., 2013). It has been widely accepted that stroke-induced damage to part of a brain network could have harmful effects on its entire function (Dijkhuizen et al., 2014). Functional MRI (fMRI) is a common imaging technique that has been used to investigate the functional change in the brains of patients with several psychiatric and neurological disorders (Sun et al., 2011). Among them, resting-state fMRI (rs-fMRI) measures spontaneous fluctuations in neural signaling, detected as low-frequency blood oxygenation level–dependent oscillations, which are synchronized among functionally connected brain regions (Dijkhuizen et al., 2014). Previous studies have applied the rs-fMRI technique, which suggested that the patients with PSCI have less functional connectivity within the brain networks (Ding et al., 2014; Dacosta-Aguayo et al., 2015).

The interventions of PSCI included controlling vascular risk factors, pharmacological treatments such as cholinesterase inhibitors (galantamine, donepezil, rivastigmine), the *N*-methyl D-aspartate antagonist memantine, and various Chinese medicines, cognitive training, and non-invasive stimulation (Beristain and Golombievski, 2015; Iadecola et al., 2019). Recently, repetitive transcranial magnetic stimulation (rTMS) is a non-invasive and relatively safe electrophysiological technique, based on the principle of electromagnetic induction of an electric field in the brain, that has been widely used in the field of rehabilitation of post-stroke dysfunctions such as motor disorder, neglect, and swallowing impairment (Winstein et al., 2016; Dionisio et al., 2018; Lefaucheur et al., 2020). Nevertheless, there are few studies about the treatment of PSCI by rTMS, and its therapeutic effects remain unclear. Rektorova et al. (2005) observed that high-frequency rTMS applied over the left DLPFC may induce positive effects on executive functioning in patients with cerebrovascular disease. However, the sample size was only seven and further studies involving larger sample sizes needed to be performed. Although some studies have not detected significant effects, others have found that the effects of rTMS treatment are not as significant as that of cognitive training, which may be related to factors such as choice of scale, rTMS parameters, and insufficient sample size (Kim et al., 2010; Park and Yoon, 2015). Therefore, the therapeutic effects of rTMS for PSCI patients remain controversial and further research is required.

In the present study, we examined the effects of rTMS on cognitive functions by expanding the sample size, modifying the rTMS parameters, and evaluating the overall cognitive function, especially the executive and memory function, and activities of daily living (ADL) that is closely related to cognition recovery. We measured two markers for resting-state fMRI including the amplitude of low-frequency fluctuations (ALFF) (0.01–0.08 Hz) of the blood oxygenation level–dependent signal used to reflect spontaneous neural activity and functional connectivity (FC) used to investigate the functional relationship between different regions at a network level (Liu et al., 2014; Lei et al., 2017; Pan et al., 2017). We expect to identify potential neural correlates to behavioral improvements following rTMS intervention on these PSCI patients.

## Materials and Methods

### Participants

**FIGURE 1:**
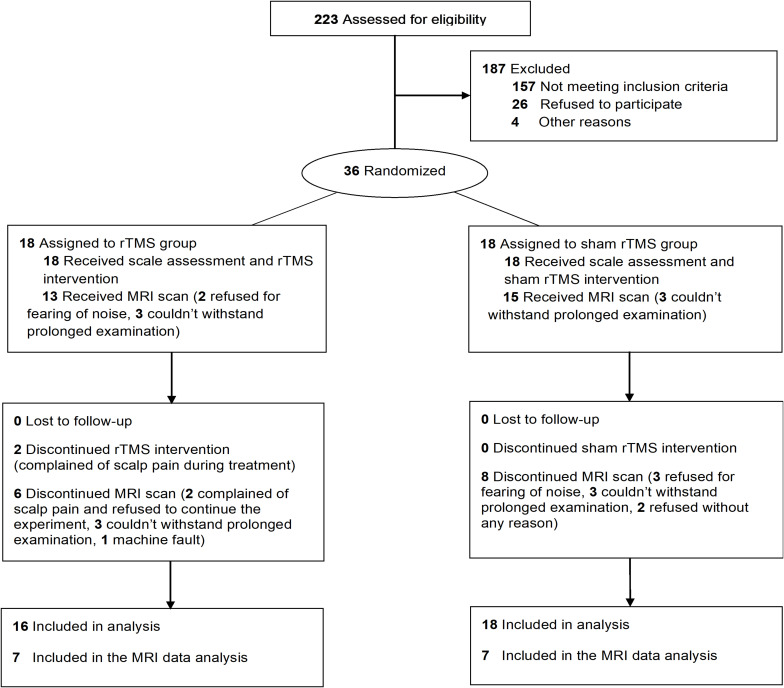
Trial flowchart.

A total of 34 PSCI patients were recruited for the present study between August 2017 and August 2019, which were subdivided for rTMS (*n* = 16) and no-stim control (*n* = 18) treatment groups using a computer-generated list of random numbers. MRI scanning was completed in seven patients in each group (Figure 1). Demographics and clinical characteristics are presented in Tables 1, 2. There was no difference between the two groups in terms of demographic and clinical characteristics.

**TABLE 1:** Baseline characteristics of all the patients with PSCI enrolled.

Variables	rTMS (*N* = 16)	No-stim control (*N* = 18)	*P*	χ^2^/ *t*/*z* value	*df*
Sex (male/female)	14/2	16/2	0.90	0.02	1
Age, mean (SD), years	56.69 (12.92)	58.17 (11.27)	0.72	−0.36	32
Type of stroke (hemorrhagic/ischemic)	5/11	6/12	0.90	0.02	1
Side of stroke (left/right/bilateral)	4/6/6	6/7/5	0.91	0.51	2
Education, mean (SD), years	10.03 (4.15)	9.33 (3.87)	0.62	0.51	32
Disease duration, median (IQR), days	52 (38.25–98.75)	55 (39.75–94.75)	0.88	−0.16	
MoCA score, mean (SD)	13.06 (6.90)	14.72 (5.76)	0.45	−0.76	32
VST-A: time, median (IQR), s	42 (35.50–63)	42.50 (33.50–51.25)	0.80	−0.26	
VST-B: time, median (IQR), s	67.50 (48.50–92.50)	48.50 (37.75–120)	0.57	−0.57	
VST-C: time, mean (SD), s	100.13 (41.38)	107.72 (61.35)	0.68	−0.42	32
VST-A: error words, median (IQR) VST-B: error words, median (IQR) VST-C: error words, mean (SD) RBMT score, mean (SD) MBI score, mean (SD)	0.50 (0–1) 1.50 (0–4) 4.94 (2.67) 7.75 (4.36) 48.06 (19.52)	1.50 (0–2) 2.50 (0–6) 5.72 (3.89) 9.33 (5.76) 50.33 (24.03)	0.12 0.46 0.50 0.38 0.77	−1.56 -0.74 -0.68 -0.90 -0.30	32 32 32

*PSCI, post-stroke cognitive impairment; rTMS, repetitive transcranial magnetic stimulation; MoCA, Montreal Cognitive Assessment Scale; VST, Victoria Stroop Test; RBMT, Rivermead Behavior Memory Test; MBI, Modified Barthel Index. Data presented as mean (SD) or median (interquartile range, IQR).*

**TABLE 2:** Baseline characteristics of PSCI patients with fMRI scan.

Variables	rTMS (*N* = 7)	No-stim control (*N* = 7)	*P*	χ^2^/ *t*/*z* value	*df*
Sex (male/female)	6/1	5/2	0.52	0.42	1
Age, median (IQR), years	58 (41–65)	62 (40–63)	1	0.00	
Type of stroke (hemorrhagic/ischemic)	3/4	3/4	1	0.00	1
Side of stroke (left/right/bilateral)	3/3/1	3/3/1	1	0.00	2
Education, median (IQR), years	12 (12–16)	11 (3–12)	0.21	−1.32	
Disease duration, mean (SD), days	84.86 (50.43)	76.14 (48.79)	0.75	0.33	12
MoCA score, mean (SD)	13.29 (7.65)	11.29 (6.50)	0.61	0.53	12
VST-A: time, mean (SD), s	42.14 (17.31)	56 (33.90)	0.35	−0.96	12
VST-B: time, median (IQR), s	61 (50–70)	38 (33–123)	0.95	−0.06	
VST-C: time, mean (SD), s	88.71 (24.62)	108.71 (61.02)	0.44	−0.80	12
VST-A: error words, median (IQR) VST-B: error words, median (IQR) VST-C: error words, mean (SD) RBMT score, mean (SD) MBI score, median (IQR)	0 (0–1) 0 (0–3) 4.14 (3.13) 7.86 (6.26) 27 (26–70)	2 (0–4) 4 (0–7) 5.86 (3.53) 7.71 (5.35) 45 (37–65)	0.24 0.42 0.36 0.96 0.48	−1.17 -0.80 -0.96 0.05 -0.70	

*PSCI, post-stroke cognitive impairment; fMRI, functional MRI; rTMS, repetitive transcranial magnetic stimulation; MoCA, Montreal Cognitive Assessment Scale; VST, Victoria Stroop Test; RBMT, Rivermead Behavior Memory Test; MBI, Modified Barthel Index. Data presented as mean (SD) or median (interquartile range, IQR).*

Written informed consent was obtained from all patients before the treatments and the study was approved by the Institutional Ethics Committee of The Third Affiliated Hospital of Sun Yat-sen University and registered at Chictr.org (Chinese Clinical Trial Registry Unique Identifier: ChiCTR-IPR-17011908, date of registration: July 8, 2017).

The inclusion criteria were as follows: (1) stroke patients in accordance with the diagnostic criteria established by the fourth National Cerebrovascular Disease Academic Conference in 1995 confirmed by a brain CT or MRI; (2) first-ever stroke, course of stroke between 1 and 6 months; (3) right-handed; (4) aged 30–75 years; (5) the presence of cognitive impairments (Montreal Cognitive Assessment, MoCA < 26); (6) no severe aphasia and able of accomplishing cognitive tests; (7) stable vital signs, no progression of neurological symptoms; (8) normal cognitive functions before stroke; (9) capable of tolerating MRI scan; (10) voluntary participation and signed the informed consent.

The exclusion criteria were as follows: (1) non-first stroke; (2) complete left prefrontal cortex injury confirmed by CT/MRI; (3) transcranial surgery or skull defect; (4) metal or cardiac pacemaker implants; (5) history of brain tumor, brain trauma, seizures, and risks of seizures; (6) cognitive function recession before stroke; (7) any neuropsychiatric comorbidity and affective disorder that could influence the test outcomes; (8) any other factors that could affect cognitive assessments and treatments.

### Outcome Measurements

The primary outcome measure, Montreal Cognitive Assessment (MoCA), was used for evaluating the general cognitive function. The secondary outcome measures were Victoria Stroop Test (VST), Rivermead Behavior Memory Test (RBMT), and Modified Barthel Index (MBI), which were used for evaluating executive function, memory, and activities of daily living (ADL). All the assessments were conducted before treatments, after 2-week treatments, and after 4-week treatments (the day after the final treatments) by an independent occupational therapist.

MoCA is widely used for evaluating cognitive functions containing seven cognitive subtests: visual-executive, naming, attention, language, abstraction, delayed recall, and orientation. One point is added for patients with or less than 12 years of schooling (Nasreddine et al., 2005).

VST, a brief version of Stroop test, is more suitable for patients with brain injury, which consists of three test cards: a colored dots trail (A), a neutral words trail (B), and an incongruent-colored words trail (C). Each card contains 24 dots/words and the outcome measures are the time consumed and the number of making errors when reading (Bayard et al., 2011; Tremblay et al., 2016).

RBMT is designed to evaluate daily memory ability, which is made up of 11 subtests: remembering names, remembering hidden belongings, remembering an appointment, picture recognition, prose recall, recalling a short route, remembering an errand, orientation, and date and face recognition (Lu et al., 2015).

MBI is used to assess the ADL including 10 subtests: personal hygiene, bathing, feeding, toileting, stair climbing, dressing, bowel control, bladder control, ambulation, or wheelchair and chair–bed transfer (Leung et al., 2007).

### rTMS Procedure

rTMS treatment was conducted with a MagPro X100 magnetic stimulators (The MagVenture Company, Denmark) and a standard figure-of-eight air-cooled coil (MCF-B65). To detect motor evoked potentials, we used the disposable ECG electrode from Xi’an fude (SEAg-J-22 × 32). Acquisition software was MAGPRO G3. Amplifier type was differential digital amplifier named Magpro MEP Monitor. Filter was high pass 20 Hz–low pass 10 kHz. The sampling rate was 100 kHz. Magnification factor was common mode rejection ratio, > 55 dB. Before stimulating, a recording electrode was attached to the contralesional first dorsal interosseous (FDI) muscle of the patients as the target muscle and the minimal stimulus intensity required to produce motor evoked potential > 50 μV in more than 5 out of 10 trials was defined as resting motor threshold (MT) (Rossi et al., 2009).

Then, 10-Hz rTMS was applied at 80% RMT, with trains of 5-s duration (50 pulses per train), 25-s inter-train interval, and a of total 40 trains (2000 pulses) costing 20 min on the left side of the DLPFC each day. We determined the optimum position for activation of the right FDI muscle by moving the coil in 0.5-cm steps around the presumed hand area of the left M1. The site at which stimuli of slightly suprathreshold intensity consistently produced the largest MEPs in the target muscle was marked as the “FDI hot spot.” For L-DLFPC stimulation, the rTMS coil was positioned 5 cm anterior to the left “FDI hot spot” (Rektorova et al., 2005). For rTMS group, the stimulating coil was placed tangentially to the surface of the skull, whereas for the no-stim control group, the coil was placed perpendicularly to the surface of the skull inducing no magnetic field. All patients received treatments once a day, 5 days per week for 4 weeks.

After rTMS treatments, patients received a 30-min computer-assisted cognitive rehabilitation referring to attention, executive function, memory, calculation, language and visuospatial skills, etc. Therapists were blinded to assignments. Besides, during hospitalization, patients received conventional drug treatments recommended by the 2016 American Heart Association/American Stroke Association recommendation (Winstein et al., 2016).

### MRI Image Acquisition and Data Pre-processing

Images were obtained before treatments and after the last rTMS treatments using a 3.0-T Discovery MR 750 Scanner (General Electric Company, United States) with an eight-channel phased array head coil. Patients were required to remain awake with their eyes closed without thinking anything. High-resolution T1-weighted structural MRI was acquired with a 3D T1-BRAVO echo sequence with parameters as follows: repetition time/echo time = 7.8 ms/3.0 ms, flip angle = 13°, field of view = 256 × 256 mm, matrix = 256 × 256, slice thickness = 1.0 mm, voxel size = 1 mm × 1 mm × 1 mm, and 176 slices. Resting fMRI was acquired using an echo-planar-imaging sequence: repetition time/echo time = 2000/30 ms, flip angle = 90°, field of view = 230 × 230 mm, matrix = 64 × 64, slices = 33, slice thickness = 3.6 mm, gap = 0.6 mm, voxel size = 3.6 mm × 3.6 mm × 3.6 mm, and total volumes = 240.

Functional images were preprocessed with the Data Processing Assistant for Resting-State fMRI software (DPARSF^1^) (Chao-Gan and Yu-Feng, 2010), which is based on Statistical Parametric Mapping 8. For each subject, the first 10 volumes were discarded to allow the mean magnetization to reach a steady state and the participants to get familiar to the MR scan environment. The remaining data were then corrected for the acquisition time difference between the 2D image slices and were realigned to the first volume to correct head motions. The maximum translational motion of all subjects was less than 3 mm and the maximum rotation was less than 3°. Images were registered with the 3D T1-weighted structural MRI and subsequently registered into the MNI standard space using the transform defined based on the registration process of the T1-weighted MRI (to the MNI space). They were then smoothed with an isotropic 3D Gaussian kernel with a full width at half maximum of 6 mm^3^. Finally, band-pass filtering (0.01 < *f* < 0.08 Hz) was performed and a linear trend was removed.

### ALFF and FC Analysis

ALFF analysis was performed using the Resting-State fMRI Data Analysis Toolkit (REST V1.8^2^) (Chao-Gan and Yu-Feng, 2010). In summary, for a given voxel, a fast Fourier transformation was used to convert the time course to the frequency domain. The mean square root, being computed and averaged throughout 0.01–0.08 Hz at each voxel, was regarded as the ALFF. Also, individual ALFF map was divided by the global mean ALFF within the mask for standardization purposes. Finally, all ALFF maps were spatially smoothed with a 6-mm full-width at half-maximum Gaussian kernel.

Regions showing significant ALFF differences after rTMS treatments were finally defined as regions of interest (ROIs), which were chosen as the seeds for FC analysis. Then, correlation analysis was performed between the seed and the whole brain in a voxel-wise manner. Finally, an entire brain *z*-value map was created after normalizing these FC values calculated from the correlation analysis by Fisher r-to-z transformation.

### Statistical Analysis

Statistical analyses were performed with SPSS V.22.0 software (IBM, Armonk, NY, United States). Shapiro–Wilk tests were used to examine the normal distribution. Two-way repeated-measures ANOVA with Bonferroni correction for *post hoc* comparisons was conducted to assess the dynamic differences within and between groups over time for clinical assessments including MoCA, VST, RBMT, and MBI scales (a log or square-root transformation was applied to achieve a normal distribution when the data were non-normally distributed). Two-sample *t*-test and non-parametric Mann–Whitney tests were used to compare two groups in terms of continuous variables, and the χ^2^ test was used for categorical variables. Pearson correlations were used to measure the association between primary outcome and secondary outcomes. Normally distributed data were expressed as mean (SD) whereas non-normally distributed data were expressed as the median (interquartile range). The significance threshold was set to *P* < 0.05. Paired *t*-test and one-sample *t-*test were performed on the maps of ALFF and FC to obtain functional differences (cluster-wise false discovery rate (FDR) corrected, voxel-level *P* < 0.005). Regions showing significant ALFF and FC differences after rTMS treatments were defined as regions of interest (ROIs). ALFF and FC values were subsequently extracted from the seed regions within each subject. Pearson correlations were calculated to measure the association between the change of neuropsychological test and the change of ALFF and FC values.

## Results

### Primary Outcome Changes

**FIGURE 2:**
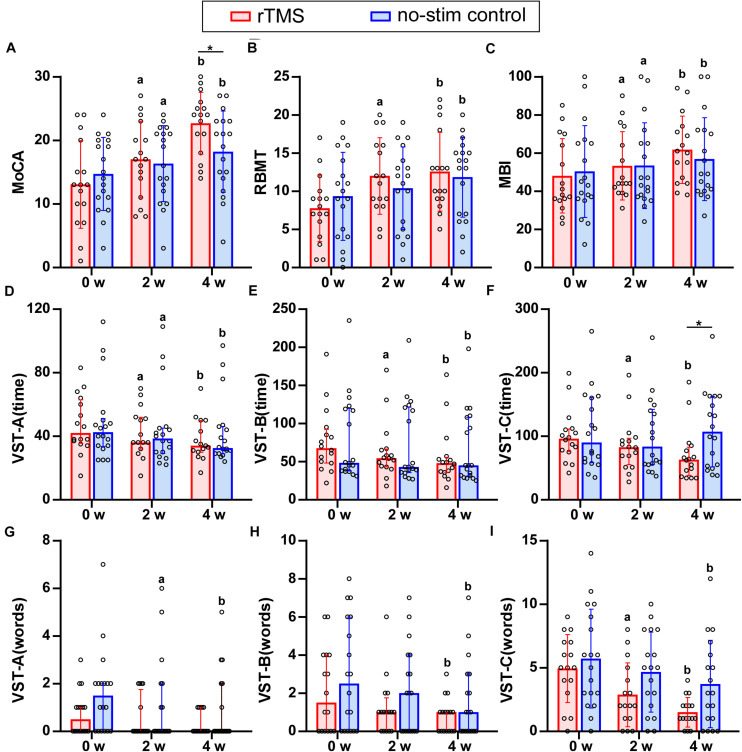
Results of neuropsychological test for rTMS group or no-stim control group. **(A)** Differences in MoCA score. **(B)** Differences in RBMT score. **(C)** Differences in MBI score. **(D–I)** Differences in the time consumed and number of making error words of VST scale. Data are analyzed by two-way repeated measures ANOVA. **(A,B,C,I)** Tops of column indicate mean and vertical lines demote SD. **(D–H)** Tops of column indicate median and vertical lines denote 25th and 75th percentiles. rTMS group is colored in red and no-stim control group is colored in blue. ^*a*^*P* < 0.05: intra-group comparison (0w vs. 2w). ^*b*^*P* < 0.05: intra-group comparison (0w vs. 4w). *P < 0.05: inter-group comparison.

For the primary outcome, two-way repeated measures ANOVA of MoCA test revealed a significant interaction effect between group and time (*F* = 17.5, *df* = 1.5, *P* < 0.001). Pairwise comparisons showed that the MoCA score in both groups increased significantly after 2 and 4 weeks (*P* < 0.05) and the score for rTMS group was significantly higher than that in the no-stim control group after 4-week treatments (*P* = 0.03) (Figure 2A).

### Secondary Outcome Changes

Referring to memory ability, two-way repeated measures ANOVA of RBMT revealed a significant interaction effect between group and time (*F* = 5.2, *df* = 2, *P* = 0.008). Pairwise comparisons showed that RBMT score increased significantly for rTMS group after 2- and 4-week treatments (*P* < 0.001), and for no-stim control group after 4-week treatment (*P* = 0.003) (Figure 2B).

Regarding the ADL, two-way repeated measures ANOVA of MBI test showed a significant interaction effect between group and time (*F* = 5.74, *df* = 1.25, *P* = 0.02). Pairwise comparisons showed that MBI scores increased significantly after 2 and 4 weeks of treatments for both groups (*P* < 0.05) (Figure 2C).

There was no significant difference in the group-by-time interaction for the two-way repeated measures ANOVA of VST-A (time) (*F* = 1.07, *df* = 1.39, *P* = 0.33), VST-A (error words) (*F* = 0.70, *df* = 2, *P* = 0.50), VST-B (error words) (*F* = 0.58, *df* = 2, *P* = 0.94), and VST-C (error words) (*F* = 2.91, *df* = 2, *P* = 0.06), whereas a significant interaction between group and time was found in VST-B (time) (*F* = 4.81, *df* = 1.29, *P* = 0.03) and VST-C (time) (*F* = 10.11, *df* = 1.21, *P* = 0.002). Pairwise comparisons revealed that the time consumed of VST-C in rTMS group was significantly lower than that in the no-stim control group after 4 weeks of treatments (*P* = 0.03) (Figures 2D–I).

### Comparison of the Improvement of Neuropsychological Test Between rTMS Group and No-Stim Control Group

**FIGURE 3:**
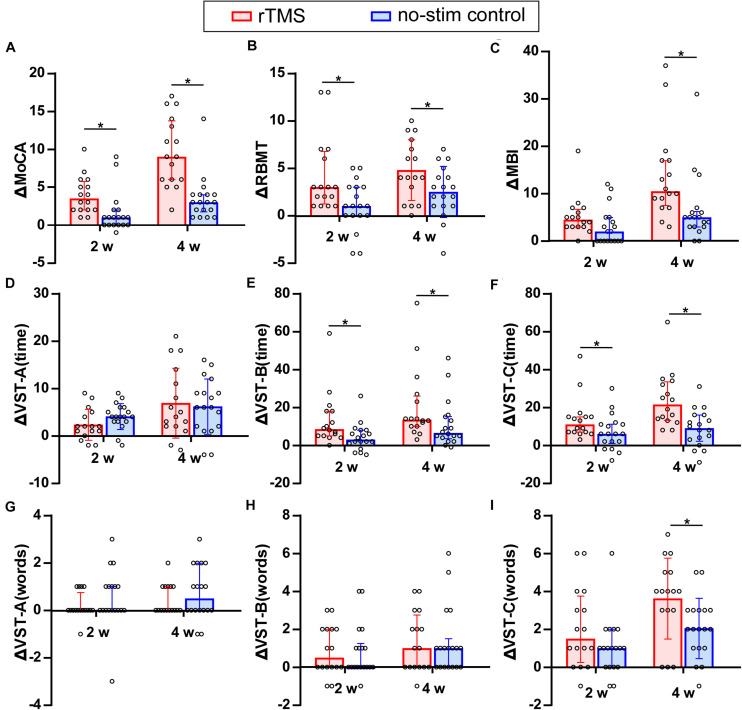
Comparison of the improvement of neuropsychological test between rTMS group and no-stim control group. **(A)** Differences in MoCA score. **(B)** Differences in RBMT score. **(C)** Differences in MBI score. **(D–I)** Differences in the time consumed and number of making error words of VST scale. The panels **(A,B-2w,C,E–H,I-2w)** are non-normally distributed data and analyzed by non-parametric Mann–Whitney *U* test. Tops of column indicate median and vertical lines demote 25th and 75th percentiles. The panels **(B-4w,D,I-4w)** are normally distributed data and analyzed by two-sample *t*-test. Tops of column indicate mean and vertical lines denote SD. rTMS group is colored in red and no-stim control group is colored in blue. *P < 0.05: inter-group comparison.

Two-sample *t*-tests were used for the analysis of VST-A (time), VST-C (error words) (4w), and RBMT (4w) and non-parametric Mann–Whitney *U* tests were used for the analysis of MoCA, RBMT (2w), and the other parts of VST test. The improvement of the MoCA score for rTMS group was significantly higher than that in the no-stim control group after 2 weeks (*Z* = −3.25, *P* = 0.001) and 4 weeks of treatments (*Z* = −4, *P* < 0.001) (Figure 3A). The improvement of the RBMT score for rTMS group was significantly higher than that in the no-stim control group after 2 weeks (*Z* = −2.46, *P* = 0.01) and 4 weeks of treatments (*F* = 1.15, *t* = 2.29, df = 32, *P* = 0.03) (Figure 3B). Moreover, the improvement of the MBI score for rTMS group was significantly higher than that in the no-stim control group after 4 weeks of treatments (*Z* = −3.08, *P* = 0.002) (Figure 3C). There was no difference between the two groups in the reduction of time consumed of VST-A (Figure 3D). The reduction of time consumed of VST-B was significantly higher than that in the no-stim control group after 2 weeks (Z = −2.56, *P* = 0.01) and 4 weeks of treatments (*Z* = −2.13, *P* = 0.03) (Figure 3E), and similar results were found in VST-C (2 weeks, *Z* = −2.17, *P* = 0.03; 4 weeks, Z = −3.18, *P* = 0.001) (Figure 3F). There was no difference between the two groups in the reduction of number of making error words of VST-A and VST-B (Figure 3G–H). The reduction of the number of making error words of VST-C for rTMS group was significantly higher than that in the no-stim control group after 4 weeks of treatments (*F* = 1.29, *t* = 2.16, df = 32, *P* = 0.04) (Figure 3I). The number of making error words of VST-C for rTMS group was also significantly higher than that in the no-stim control group after 4 weeks of treatments (*F* = 1.29, *t* = 2.16, df = 32, *P* = 0.04) (Figure 3I).

### Changes of MoCA Test for Sub-Items

**FIGURE 4:**
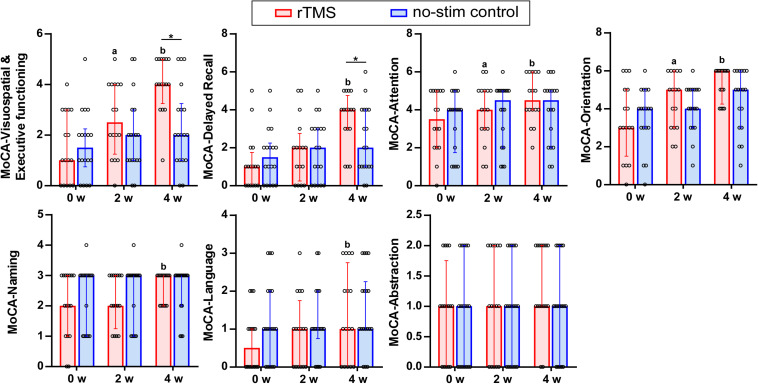
Sub-items of the MOCA test in detail. Data are analyzed by two-way repeated measures ANOVA. Tops of column indicate median and vertical lines demote 25th and 75th percentiles. rTMS group is colored in red and no-stim control group is colored in blue. ^*a*^*P* < 0.05: intra-group comparison (0w vs. 2w). ^*b*^*P* < 0.05: intra-group comparison (0w vs. 4w). *P < 0.05: inter-group comparison.

By using two-way repeated measures ANOVA for analyzing the seven sub-items of the MoCA test, significant interaction effects between group and time were found in the cognitive domain of visuospatial and executive functioning (*F* = 20.27, *df* = 1.49, *P* = 0.001), attention (*F* = 4.42, *df* = 1.66, *P* = 0.02), and delayed recall (*F* = 6.90, *df* = 2, *P* = 0.002). Pairwise comparisons showed that rTMS group differed significantly from no-stim control group in executive functioning (*P* = 0.003) as well as delayed recall (*P* = 0.04) (Figure 4).

### Correlation of Changes for the Primary Outcome and the Secondary Outcomes

**FIGURE 5:**
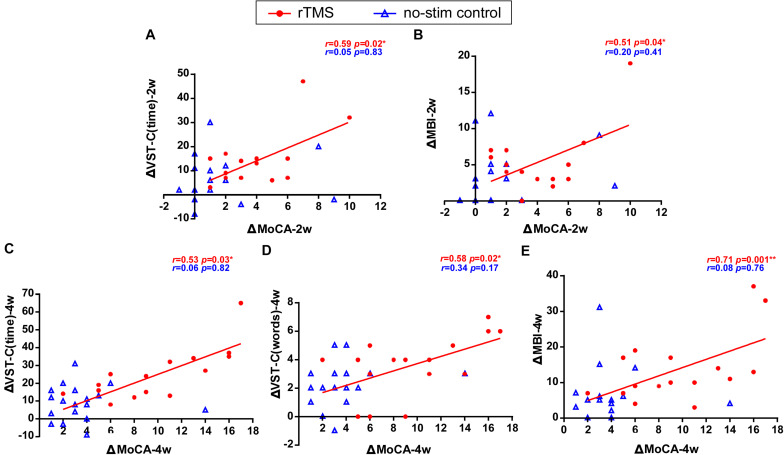
Correlation of changes for the primary outcome and the secondary outcomes. Data are analyzed by Pearson correlations. **(A)** The improvement of the score of MoCA test positively correlated with the reduction of the time consumed of VST-C after 2 weeks of rTMS treatments. **(B)** The improvement of the score of MoCA test positively correlated with the improvement of the score of MBI test after 2 weeks of rTMS treatments. **(C)** The improvement of the score of MoCA test positively correlated with the reduction of the time consumed of VST-C after 4 weeks of rTMS treatments. **(D)** The improvement of the score of MoCA test positively correlated with the reduction of the number of making error words of VST-C after 4 weeks of rTMS treatments. **(E)** The improvement of the score of MoCA test positively correlated with the improvement of the score of MBI test after 4 weeks of rTMS treatments.

The correlation results showed that the change of the scores of MoCA test after 2 weeks of rTMS treatments positively correlated with the reduction of time consumed of VST-C test (*r* = 0.59, *P* = 0.02) (Figure 5A) and the improvement of the scores of MBI test (*r* = 0.51, *P* = 0.04) (Figure 5B). Besides, after 4 weeks of rTMS treatments, the change of scores of MoCA test positively correlated with the reduction of time consumed of VST-C test (*r* = 0.53, *P* = 0.03) (Figure 5C), the reduction of numbers of making error words of VST-C test (*r* = 0.58, *P* = 0.02) (Figure 5D), and the improvement of the scores of MBI test (*r* = 0.71, *P* = 0.001) (Figure 5E).

### Effects of rTMS Treatment on Neural Activity and Connectivity

**FIGURE 6:**
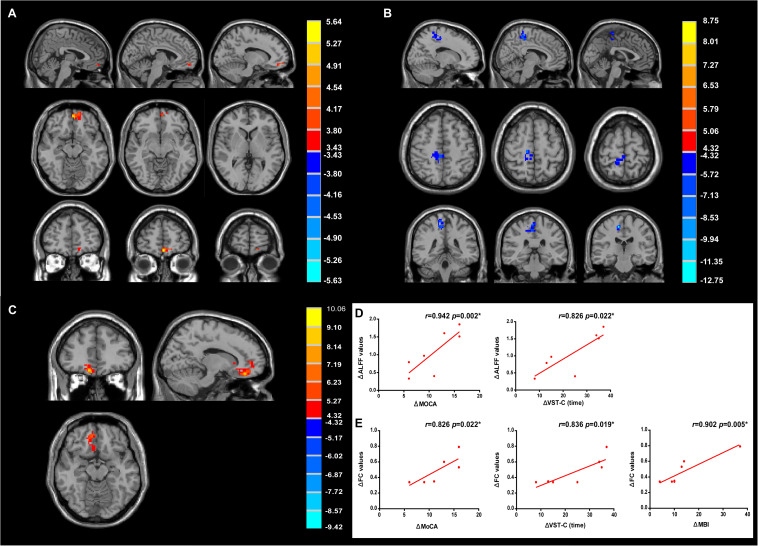
Modulation of amplitude of low-frequency fluctuation (ALFF) and functional connectivity (FC) after treatments. **(A)** Change of ALFF in rTMS group. **(B)** Change of ALFF in no-stim control group. **(C)** Increased FC in rTMS group. Significance level was defined at *P* < 0.005, cluster size > 51 voxels. FDR corrected. The left side of the image corresponds to the right side of the brain. **(D)** The improvement of MoCA and VST-C test positively correlated with the change of ALFF values. **(E)** The improvement of MoCA, VST-C, and MBI test positively correlated with the change of FC values.

With 10 Hz left DLPFC rTMS treatments, ALFF in the left medial prefrontal cortex (peak MNI coordinates: *x* = −5, *y* = 57, *z* = −15, *P* < 0.005, *t* = 5.64, voxels = 53, corrected by FDR) (Figure 6A and Table 3) was significantly increased, whereas for no-stim control treated group, significantly decreased ALFF was found in the right posterior parietal cortex (peak MNI coordinates: *x* = 12, *y* = −24, *z* = 57, *P* < 0.005, *t* = −12.75, voxels = 135, corrected by FDR) (Figure 6B and Table 3).

**TABLE 3:** Changes of ALFF and FC in rTMS group and no-stim control group after 4-week treatments.

Brain regions	Brodmann’s area	MNI coordinates			Peak *t*-value	Voxels
		x	y	z		
**ALFF**						
rTMS (4w > baseline)						
L-MPFC	10	−5	57	−15	5.64	53
No-stim control (4w > baseline)						
R-PPC	6	12	−24	57	−12.75	135
**FC**						
R-MPFC	10	9	45	−18	7.19	69
R-vACC	25	10	28	−14	10.06	56

*ALFF, amplitude of low-frequency fluctuation; FC, functional connectivity; MNI, Montreal Neurological Institute coordinates; L, left; R, right; MPFC, medial prefrontal cortex; PPC, posterior parietal cortex; vACC, right ventral anterior cingulate cortex.*

Further FC analysis was based on the ROIs (the regions showing significant ALFF differences after rTMS treatments), and the results showed that FC increased significantly in the right medial prefrontal cortex (peak MNI coordinates: *x* = 9, *y* = 45, *z* = −18, *P* < 0.005, *t* = 7.19, voxels = 69, corrected by FDR) and the right ventral anterior cingulate cortex (vACC) (peak MNI coordinates: *x* = 10, *y* = 28, *z* = −14, *P* < 0.005, *t* = 10.06, voxels = 56, corrected by FDR) (Figure 6C and Table 3).

Correlation analyses revealed that the change of the ALFF values of the left medial prefrontal cortex positively correlated with the improvement of the score of MoCA test (*r* = 0.942, *P* = 0.002) and the reduction of time consumed of VST-C (*r* = 0.826, *P* = 0.022) after rTMS treatments (Figure 6D). In addition, positive correlation between the change of the FC values of the right medial prefrontal cortex, vACC, and the improvement of MoCA (*r* = 0.826, *P* = 0.022), VST-C (*r* = 0.836, *P* = 0.019), and MBI test (*r* = 0.902, *P* = 0.005) after rTMS treatments were identified (Figure 6E).

## Discussion

The present study reported the effects of chronic high-frequency rTMS at DLPFC on the improvement of cognitive function and life quality in PSCI patients. These changes accompanied altered activation of local frontal regions and connectivity changes within the frontal network. These results demonstrated that DLPFC rTMS treatment is tolerable, therapeutically effective for PSCI patients, and warrants larger clinical trials in future studies.

We found that rTMS at DLPFC improved executive function for PSCI patients. The results were in accordance with a previous study reporting that TMS improves executive function for patients of cerebrovascular disease with cognitive impairment (Rektorova et al., 2005). In addition, the memory improvement was inconsistent with a previous study using 1-Hz rTMS at the right DLPFC for stroke patients (Lu et al., 2015), in which they reported peripheral brain-derived neurotrophic factor (BDNF) changes as well. It is undetermined if peripheral biomarkers (e.g., BDNF) could predict the clinical improvements for the cognitive function of these patients. One study found that rTMS treatment at DLPFC improves emotion but not cognitive function for PSCI patients (Kim et al., 2010). It is undeniable that rTMS could improve mood disorder for patients with PSCI. However, the study was not sufficiently powered to detect positive effects on cognition and may likely be a result of their incomplete scale, stimulus parameters, and small sample size. Furthermore, cognitive dysfunction had a strong impact on the ADL (Paker et al., 2010). Cognitive functioning early after stroke was found to be an independent predictor of long-term functional outcomes of ADL (McKinney et al., 2002). Our results showed that a higher MoCA score was associated with better performance on MBI score. Irfani et al. (2020) found a positive association between cognitive function and ADL in post-stroke patients and the improvement of ADL may be a result of the recovery of working memory, referring to maintain and manipulate information, which is processed by the frontal lobe. Liu et al. (2020) have also demonstrated that TMS could improve the performance in the ADL and attention function in patients with stroke, which is in line with our study.

The reason we chose DLPFC as a stimulus site is that the DLPFC is related to cognitive function mainly for processing speed, selective attention, working memory, and episodic memory (Gaudeau-Bosma et al., 2013; Parkin et al., 2015; Alcala-Lozano et al., 2017). Besides, DLPFC plays an important part in the central executive network (CEN), which is responsible for high-level cognitive functions, notably the control of attention and working memory (Bressler and Menon, 2010). A meta-analysis has shown that high-frequency rTMS stimulation of DLPFC could improve the cognitive function for healthy people, patients with Alzheimer’s disease, depression, executive dysfunction, memory complaints, and Parkinson’s disease (Guse et al., 2010). It is recommended that rTMS at 10, 15, or 20 Hz be applied over the left DLPFC within an individual motor threshold of 80–110%, which is most likely to cause significant cognitive improvement (Guse et al., 2010). We did not completely copy the rTMS parameters of previous studies on rTMS treatment of PSCI and made some modifications. The reason why we increased the total number of stimuli to 2,000 was to consider the possibility that increasing the number of stimuli might increase the efficacy and duration of rTMS treatments. A systematic review also concluded that long-term cognitive improvements are likely related to the number of stimulation sessions/days, where more stimulation sessions result in longer-lasting stimulation effects (van Lieshout et al., 2019).

Previous studies reported that patients with PSCI showed less functional connectivity within the brain network (Ding et al., 2014; Dacosta-Aguayo et al., 2015), especially the default-mode network (DMN), which consists of the medial prefrontal cortex (MPFC), posterior cingulate cortex, precuneus, hippocampus, inferior temporal cortex, and inferior parietal lobules (Andrews-Hanna et al., 2010). Stroke patients exhibited decreased functional connectivity in the medial prefrontal, left medial temporal lobe, and posterior cingulate cortex within the DMN, which potentially contributes to the cognitive dysfunction in stroke patients (Tuladhar et al., 2013). PSCI patients showed decreased functional connectivity in the MPFC and hippocampus than stroke patients without cognitive impairment (Ding et al., 2014). Moreover, the increased local activity of MPFC during natural recovery may reflect compensation for loss of cognitive function in stroke patients because MPFC was related to executive functions, task processes, emotional regulation, and social-cognitive processes that are related to the self and others (Ding et al., 2014). It is conceivable that rTMS treatment may facilitate the compensation for cognitive functions after stroke. Besides, Tang et al. (2019) have observed that cognitive training could improve the global cognitive function of patients with subcortical vascular cognitive impairment or dementia and significantly increased the connectivity between the left DLPFC and MPFC, indicating that the connection between DMN and CEN was important in the recovery of cognitive function. Referring to our study, although the stimulation site was DLPFC, the neural activation was found in MPFC, which could be attributable to the connection of networks.

Our subsequent FC analysis revealed enhanced right MPFC and vACC FC to the left MPFC, which further explained the important role of MPFC in the recovery of cognitive function for stroke patients. The vACC is associated with decision-making, emotion regulation, and self-referential processing and is an important part of the salience network, mediating the conversion of the functional connectivity between the DMN and CEN (Hamilton et al., 2011; van den Heuvel et al., 2013; Yoshimura et al., 2014). Yoshimura et al. (2014) found that cognitive-behavioral therapy influenced MPFC and vACC functioning related to self-referential processing for depression patients. Xue et al. (2017) reported a similar conclusion with our study and found an increased fractional amplitude of low-frequency fluctuation in rostral ACC (one part of vACC) after 20 Hz rTMS on the left DLPFC for healthy participants. Besides, they observed an enhanced rACC/vmPFC FC to frontal, temporal, and hippocampus regions suggesting rACC as a hub region for facilitating the left DLPFC rTMS effects on DMN areas through the frontal-cingulate pathway (Xue et al., 2017). Similar to our study, they also applied high-frequency stimulation of the left DLPFC to observe changes in brain activation and functional connectivity. The differences were that they observed the immediate effects (before and after 48 h after once stimulation) and we observed the cumulative effects (before and after 4 weeks of multiple stimulations). As for our study, we suggested that the left DLPFC rTMS may activate the left MPFC and enhanced the functional connectivity to the contralateral MPFC and vACC to regulate the DMN.

This study has some limitations. First, the sample size was relatively small and only two female patients were included in each group. It is undeniable that the findings were more suitable explaining for male patients. In future studies, we will further expand the sample size to avoid this gender bias. Second, this study applied only one parameter for the rTMS treatments and failed to screen for the optimal stimulation protocol. Third, because of the lack of follow-up assessment, the long-term efficacy and sustained changes in brain function after the cessation of rTMS treatment were not determined.

In summary, our results suggest that high-frequency rTMS applied on the left DLPFC could improve cognitive function for stroke patients with cognitive impairment, with accompanying changes in the left medial prefrontal cortex.

## Data Availability Statement

All datasets presented in this study are included in the article/supplementary material.

## Ethics Statement

The studies involving human participants were reviewed and approved by the Institutional Ethics Committee of The Third Affiliated Hospital of Sun Yat-sen University. The patients/participants provided their written informed consent to participate in this study.

## Author Contributions

All authors listed have made a substantial, direct and intellectual contribution to the work, and approved it for publication.

## Conflict of Interest

The authors declare that the research was conducted in the absence of any commercial or financial relationships that could be construed as a potential conflict of interest.

## Footnotes

1 http://www.rfmri.org/DPARSF

2 http://restfmri.net/forum/REST

## References

[B1] Alcala-Lozano R. Morelos-Santana E. Cortes-Sotres J. F. Garza-Villarreal E. A. Sosa-Ortiz A. L. Gonzalez-Olvera J. J. (2017). Similar clinical improvement and maintenance after rTMS at 5 Hz using a simple vs. complex protocol in Alzheimer’s disease. *Brain Stimul.* 11 625–627. 10.1016/j.brs.2017.12.011 29326021

[B2] Andrews-Hanna J. R. Reidler J. S. Sepulcre J. Poulin R. Buckner R. L. (2010). Functional-anatomic fractionation of the brain’s default network. *Neuron* 65 550–562. 10.1016/j.neuron.2010.02.005 20188659 PMC2848443

[B3] Bayard S. Erkes J. Moroni C. (2011). Victoria Stroop Test: normative data in a sample group of older people and the study of their clinical applications in the assessment of inhibition in Alzheimer’s disease. *Arch. Clin. Neuropsychol.* 26 653–661. 10.1093/arclin/acr053 21873625

[B4] Beristain X. Golombievski E. (2015). Pharmacotherapy to enhance cognitive and motor recovery following stroke. *Drugs Aging* 32 765–772. 10.1007/s40266-015-0299-0 26423272

[B5] Bressler S. L. Menon V. (2010). Large-scale brain networks in cognition: emerging methods and principles. *Trends Cogn. Sci.* 14 277–290. 10.1016/j.tics.2010.04.004 20493761

[B6] Chao-Gan Y. Yu-Feng Z. (2010). DPARSF: a MATLAB toolbox for “Pipeline” data analysis of resting-state fMRI. *Front. Syst. Neurosci.* 4:13. 10.3389/fnsys.2010.00013 20577591 PMC2889691

[B7] Dacosta-Aguayo R. Grana M. Iturria-Medina Y. Fernandez-Andujar M. Lopez-Cancio E. Caceres C. (2015). Impairment of functional integration of the default mode network correlates with cognitive outcome at three months after stroke. *Hum. Brain Mapp.* 36 577–590. 10.1002/hbm.22648 25324040 PMC4312977

[B8] Dijkhuizen R. M. Zaharchuk G. Otte W. M. (2014). Assessment and modulation of resting-state neural networks after stroke. *Curr. Opin. Neurol.* 27 637–643. 10.1097/wco.0000000000000150 25333606

[B9] Ding X. Li C. Y. Wang Q. S. Du F. Z. Ke Z. W. Peng F. (2014). Patterns in default-mode network connectivity for determining outcomes in cognitive function in acute stroke patients. *Neuroscience* 277 637–646. 10.1016/j.neuroscience.2014.07.060 25090922

[B10] Dionisio A. Duarte I. C. Patricio M. Castelo-Branco M. (2018). Transcranial magnetic stimulation as an intervention tool to recover from language, swallowing and attentional deficits after stroke: a systematic review. *Cerebrovasc. Dis.* 46 178–185. 10.1159/000494213 30343304

[B11] Gaudeau-Bosma C. Moulier V. Allard A. C. Sidhoumi D. Bouaziz N. Braha S. (2013). Effect of two weeks of rTMS on brain activity in healthy subjects during an n-back task: a randomized double blind study. *Brain Stimul.* 6 569–575. 10.1016/j.brs.2012.10.009 23194830

[B12] Guse B. Falkai P. Wobrock T. (2010). Cognitive effects of high-frequency repetitive transcranial magnetic stimulation: a systematic review. *J. Neural Trans.* 117 105–122. 10.1007/s00702-009-0333-7 19859782 PMC3085788

[B13] Hamilton J. P. Glover G. H. Hsu J. J. Johnson R. F. Gotlib I. H. (2011). Modulation of subgenual anterior cingulate cortex activity with real-time neurofeedback. *Hum. Brain Mapp.* 32 22–31. 10.1002/hbm.20997 21157877 PMC3049174

[B14] Iadecola C. Duering M. Hachinski V. Joutel A. Pendlebury S. T. Schneider J. A. (2019). Vascular cognitive impairment and dementia: JACC scientific expert panel. *J. Am. Coll. Cardiol.* 73 3326–3344. 31248555 10.1016/j.jacc.2019.04.034 PMC6719789

[B15] Irfani Fitri F. Fithrie A. Rambe A. S. (2020). Association between working memory impairment and activities of daily living in post-stroke patients. *Med. Glasnik* 17 433–438. 10.17392/1135-20 32489085

[B16] Kim B. R. Kim D. Y. Chun M. H. Yi J. H. Kwon J. S. (2010). Effect of repetitive transcranial magnetic stimulation on cognition and mood in stroke patients: a double-blind, sham-controlled trial. *Am. J. Phys. Med. Rehabil.* 89 362–368. 10.1097/phm.0b013e3181d8a5b1 20407301

[B17] Lefaucheur J. P. Aleman A. Baeken C. Benninger D. H. Brunelin J. Di Lazzaro V. (2020). Evidence-based guidelines on the therapeutic use of repetitive transcranial magnetic stimulation (rTMS): an update (2014-2018). *Clin. Neurophysiol.* 131 474–528. 31901449 10.1016/j.clinph.2019.11.002

[B18] Lei X. Zhong M. Liu Y. Jin X. Zhou Q. Xi C. (2017). A resting-state fMRI study in borderline personality disorder combining amplitude of low frequency fluctuation, regional homogeneity and seed based functional connectivity. *J. Affect. Disord.* 218 299–305. 10.1016/j.jad.2017.04.067 28478359

[B19] Leung S. O. Chan C. C. Shah S. (2007). Development of a Chinese version of the Modified Barthel Index– validity and reliability. *Clin Rehabil* 21 912–922. 10.1177/0269215507077286 17981850

[B20] Leys D. Henon H. Mackowiak-Cordoliani M. A. Pasquier F. (2005). Poststroke dementia. *Lancet Neurol.* 4 752–759. 16239182 10.1016/S1474-4422(05)70221-0

[B21] Liu J. Qin W. Wang H. Zhang J. Xue R. Zhang X. (2014). Altered spontaneous activity in the default-mode network and cognitive decline in chronic subcortical stroke. *J. Neurol. Sci.* 347 193–198. 10.1016/j.jns.2014.08.049 25304057

[B22] Liu Y. Yin M. Luo J. Huang L. Zhang S. Pan C. (2020). Effects of transcranial magnetic stimulation on the performance of the activities of daily living and attention function after stroke: a randomized controlled trial. *Clin. Rehabil.* 4:269215520946386. 10.1177/0269215520946386 32748630

[B23] Lu H. Zhang T. Wen M. Sun L. (2015). Impact of repetitive transcranial magnetic stimulation on post-stroke dysmnesia and the role of BDNF Val66Met SNP. *Med. Sci. Monit.* 21 761–768. 10.12659/msm.892337 25770310 PMC4370352

[B24] McKinney M. Blake H. Treece K. A. Lincoln N. B. Playford E. D. Gladman J. R. (2002). Evaluation of cognitive assessment in stroke rehabilitation. *Clin. Rehabil.* 16 129–136. 10.1191/0269215502cr479oa 11926175

[B25] Nasreddine Z. S. Phillips N. A. Bedirian V. Charbonneau S. Whitehead V. Collin I. (2005). The montreal cognitive assessment, MoCA: a brief screening tool for mild cognitive impairment. *J. Am. Geriatr. Soc.* 53 695–699. 10.1111/j.1532-5415.2005.53221.x 15817019

[B26] Paker N. Buðdaycı D. Tekdö?? D. Kaya B. Dere C. (2010). Impact of cognitive impairment on functional outcome in stroke. *Stroke Res. Treat.* 2010:652612. 10.4061/2010/652612 PMC2925089 20798755

[B27] Pan P. Zhu L. Yu T. Shi H. Zhang B. Qin R. (2017). Aberrant spontaneous low-frequency brain activity in amnestic mild cognitive impairment: a meta-analysis of resting-state fMRI studies. *Ageing Res. Rev.* 35 12–21. 10.1016/j.arr.2016.12.001 28017880

[B28] Park I. S. Yoon J. G. (2015). The effect of computer-assisted cognitive rehabilitation and repetitive transcranial magnetic stimulation on cognitive function for stroke patients. *J. Phys. Ther. Sci.* 27 773–776. 10.1589/jpts.27.773 25931728 PMC4395712

[B29] Parkin B. L. Ekhtiari H. Walsh V. F. (2015). Non-invasive human brain stimulation in cognitive neuroscience: a primer. *Neuron* 87 932–945. 10.1016/j.neuron.2015.07.032 26335641

[B30] Rektorova I. Megova S. Bares M. Rektor I. (2005). Cognitive functioning after repetitive transcranial magnetic stimulation in patients with cerebrovascular disease without dementia: a pilot study of seven patients. *J. Neurol. Sci.* 229 157–161. 10.1016/j.jns.2004.11.021 15760635

[B31] Rossi S. Hallett M. Rossini P. M. Pascual-Leone A. The Safety of Tms Consensus Group. (2009). Safety of, Safety, ethical considerations, and application guidelines for the use of transcranial magnetic stimulation in clinical practice and research. *Clin. Neurophys.* 120 2008–2039. 10.1016/j.clinph.2009.08.016 PMC3260536 19833552

[B32] Sachdev P. S. Brodaty H. Valenzuela M. J. Lorentz L. Looi J. C. Berman K. (2006). Clinical determinants of dementia and mild cognitive impairment following ischaemic stroke: the sydney stroke study. *Dement. Geriatr. Cogn. Disord.* 21 275–283. 10.1159/000091434 16484805

[B33] Sun Y. W. Qin L. D. Zhou Y. Xu Q. Qian L. J. Tao J. (2011). Abnormal functional connectivity in patients with vascular cognitive impairment, no dementia: a resting-state functional magnetic resonance imaging study. *Behav. Brain Res.* 223 388–394. 10.1016/j.bbr.2011.05.006 21605598

[B34] Tang Y. Xing Y. Zhu Z. He Y. Li F. Yang J. (2019). The effects of 7-week cognitive training in patients with vascular cognitive impairment, no dementia (the Cog-VACCINE study): a randomized controlled trial. *J. Alzheimers Assoc.* 15 605–614. 10.1016/j.jalz.2019.01.009 30894299

[B35] Tremblay M. P. Potvin O. Belleville S. Bier N. Gagnon L. Blanchet S. (2016). The victoria stroop test: normative data in quebec-french adults and elderly. *Arch. Clin. Neuropsychol.* 31 926–933. 27246959 10.1093/arclin/acw029 PMC5859918

[B36] Tuladhar A. M. Snaphaan L. Shumskaya E. Rijpkema M. Fernandez G. Norris D. G. (2013). Default mode network connectivity in stroke patients. *PLoS One* 8:e66556. 10.1371/journal.pone.0066556 23824302 PMC3688936

[B37] van den Heuvel O. A. Van Gorsel H. C. Veltman D. J. Van Der Werf Y. D. (2013). Impairment of executive performance after transcranial magnetic modulation of the left dorsal frontal-striatal circuit. *Hum. Brain Mapp.* 34 347–355. 10.1002/hbm.21443 22076808 PMC6870447

[B38] van Lieshout E. C. C. van Hooijdonk R. F. Dijkhuizen R. M. Visser-Meily J. M. A. Nijboer T. C. W. (2019). The effect of noninvasive brain stimulation on poststroke cognitive function: a systematic review. *Neurorehabil. Neural Rep.* 33 355–374. 10.1177/1545968319834900 31021702

[B39] Winstein C. J. Stein J. Arena R. Bates B. Cherney L. R. Cramer S. C. (2016). Guidelines for adult stroke rehabilitation and recovery: a guideline for healthcare professionals from the american heart association/american stroke association. *Stroke* 47 e98–e169. 27145936 10.1161/STR.0000000000000098

[B40] Xue S. W. Guo Y. Peng W. Zhang J. Chang D. Zang Y. F. (2017). Increased low-frequency resting-state brain activity by high-frequency repetitive tms on the left dorsolateral prefrontal cortex. *Front. Psychol.* 8:2266. 10.3389/fpsyg.2017.02266 29312097 PMC5744634

[B41] Yoshimura S. Okamoto Y. Onoda K. Matsunaga M. Okada G. Kunisato Y. (2014). Cognitive behavioral therapy for depression changes medial prefrontal and ventral anterior cingulate cortex activity associated with self-referential processing. *Soc. Cogn. Affect. Neurosci.* 9 487–493. 10.1093/scan/nst009 23327934 PMC3989129

